# Cellular Consequences of Diminished Protein *O*-Mannosyltransferase Activity in Baker’s Yeast

**DOI:** 10.3390/ijms18061226

**Published:** 2017-06-09

**Authors:** Ewa Zatorska, Lihi Gal, Jaro Schmitt, Daniela Bausewein, Maya Schuldiner, Sabine Strahl

**Affiliations:** 1 Centre for Organismal Studies (COS), Heidelberg University, 69120 Heidelberg, Germany; ewa.zatorska@cos.uni-heidelberg.de (E.Z.); JaroSchmitt@web.de (J.S.); daniela.bausewein@cos.uni-heidelberg.de (D.B.); 2 Department of Molecular Genetics, Weizmann Institute of Science, 7610001 Rehovot, Israel; lihi.gal@weizmann.ac.il (L.G.); maya.schuldiner@weizmann.ac.il (M.S.)

**Keywords:** glycosylation, mannosyltransferase, oligosaccharyltransferase, cell wall, unfolded protein response, calcineurin, endoplasmic reticulum, GPI anchor

## Abstract

*O*-Mannosylation is a type of protein glycosylation initiated in the endoplasmic reticulum (ER) by the protein *O*-mannosyltransferase (PMT) family. Despite the vital role of *O*-mannosylation, its molecular functions and regulation are not fully characterized. To further explore the cellular impact of protein *O*-mannosylation, we performed a genome-wide screen to identify *Saccharomyces cerevisiae* mutants with increased sensitivity towards the PMT-specific inhibitor compound R3A-5a. We identified the cell wall and the ER as the cell compartments affected most upon PMT inhibition. Especially mutants with defects in *N*-glycosylation, biosynthesis of glycosylphosphatidylinositol-anchored proteins and cell wall β-1,6-glucan showed impaired growth when *O*-mannosylation became limiting. Signaling pathways that counteract cell wall defects and unbalanced ER homeostasis, namely the cell wall integrity pathway and the unfolded protein response, were highly crucial for the cell growth. Moreover, among the most affected mutants, we identified Ost3, one of two homologous subunits of the oligosaccharyltransferase complexes involved in *N*-glycosylation, suggesting a functional link between the two pathways. Indeed, we identified Pmt2 as a substrate for Ost3 suggesting that the reduced function of Pmt2 in the absence of *N*-glycosylation promoted sensitivity to the drug. Interestingly, even though *S. cerevisiae* Pmt1 and Pmt2 proteins are highly similar on the sequence, as well as the structural level and act as a complex, we identified only Pmt2, but not Pmt1, as an Ost3-specific substrate protein.

## 1. Introduction

*O*-Mannosylation is a type of protein glycosylation that is highly conserved among eukaryotes. *O*-Mannosyl glycans are crucial for the viability of yeast [1,2]. In humans, impaired *O*-mannosylation causes congenital disorders, which clinically manifest as muscular dystrophy combined with severe malformations of the eye and brain (reviewed in [3]).

Biosynthesis of *O*-mannosyl glycans is initiated in the endoplasmic reticulum (ER) where mannose is transferred from dolichol phosphate-activated mannose (Dol-P-Man) to serine and threonine residues of protein substrates (reviewed in [4]). This reaction is conserved between the fungal and animal kingdom and is catalyzed by a family of enzymes named protein *O*-mannosyltransferases (PMTs). In baker’s yeast, *Saccharomyces cerevisiae* (simply referred to as yeast), PMTs act on substrate proteins during their translocation into the ER lumen [5]. At the translocon, PMTs and the oligosaccharyltransferase (OST) complex, which catalyzes en bloc transfer of *N*-glycan precursors to nascent polypeptide chains, can compete for glycan acceptor sites [5,6,7]. Properly folded glycoproteins are transported from the ER to the Golgi apparatus where the initial mannose is further elongated in a stepwise fashion. In yeast, various α-1,2- and α-1,3- mannosyltransferases contribute to this process using guanosine diphosphate-activated mannose (GDP-mannose) as a carbohydrate donor. In this way, short linear oligomannose chains arise.

PMTs are integral ER membrane proteins with seven transmembrane domains, a cytoplasmic N-terminus and the C-terminus facing the ER lumen [8]. In yeast, the PMT family consists of at least six proteins (Pmt1-6), grouped by phylogenetic analysis into three subfamilies: PMT1 (Pmt1, Pmt5), PMT2 (Pmt2, Pmt3, Pmt6) and PMT4 (Pmt4). The single PMT4 subfamily member forms homomeric complexes, while members of the PMT1 and PMT2 subfamilies form heteromeric complexes [9]. In wild-type yeast, the majority of the enzymatic activity is due to Pmt1-Pmt2 and Pmt4-Pmt4 complexes [10,11].

PMTs play a crucial role in the maintenance of the cell wall. Most of the characterized viable and conditional lethal *pmt*Δ mutants show various cell wall-related phenotypes [1,12] and PMT-specific inhibition results in transcriptional activation of the cell wall integrity pathway (CWIP) [13]. This pathway orchestrates changes to the cell wall periodically during the cell cycle, in response to mating pheromone and cell wall stress caused by environmental factors (reviewed in [14]). A recent glycoproteomic study showed that *O*-mannosylated proteins are clearly overrepresented in the cell wall compartment, comprising more than two-thirds of all known cell wall proteins [7]. In yeast, cell wall glycoproteins, which carry *N*- and/or *O*-linked glycans and often a glycosylphosphatidylinositol (GPI) anchor remnant, contribute ~40% of the cell wall dry weight. The remaining cell wall components are chitin (~1.5%) and β-glucan (~60%) [15].

In addition to their function for cell wall stability, yeast Pmt1 and Pmt2 were suggested to play a role in ER protein quality control. Transcription of PMTs is enhanced during ER stress conditions [16], and Pmt1-Pmt2 complexes physically interact with ER folding helpers and components of the ER-associated degradation pathway [17]. Furthermore, several lines of evidence suggest that *O*-mannosylation increases the solubility of misfolded proteins, thereby reducing the chaperone load in the ER (reviewed in [18]). More recently, it was shown that an ER-targeted, poorly-folding variant of green fluorescent protein (GFP) becomes *O*-mannosylated by Pmt1-Pmt2 complexes, preventing it from achieving its final conformation [19]. It was suggested that *O*-mannosyl glycans terminate futile folding attempts of proteins in the ER. The involvement of Pmt1-Pmt2 in ER protein quality control is referred to as unfolded protein *O*-mannosylation [19].

Despite the vital role of PMTs, their regulation and full range of functions remain elusive. To further elucidate these issues, here we screened collections of *S. cerevisiae* mutants for strains with no or reduced growth when *O*-mannosylation was impaired using a chemical inhibitor.

## 2. Results

### 2.1. Systematic Analysis of Factors Important for Normal Cell Growth upon Impaired Protein O-Mannosylation

**Figure 1:**
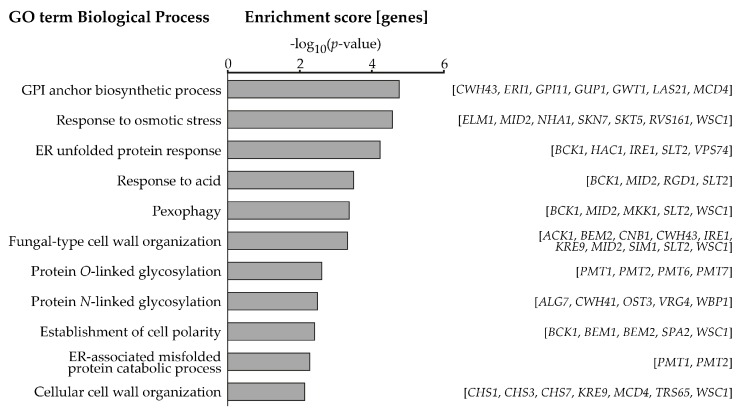
Functional categories enriched (*p*-value < 0.01) among mutants hypersensitive to protein *O*-mannosyltransferase-inhibitor R3A-5a. Gene ontology (GO) term analysis was performed using GeneCodis applying default settings. Genes assigned to the respective biological process are listed in square brackets. GPI—glycosylphosphatidylinositol. ER—endoplasmic reticulum.

We screened genome-wide collections of *S. cerevisiae* mutants for increased sensitivity towards the PMT-specific inhibitor R3A-5a. This compound is a rhodanine-3-acetic acid derivative, which blocks enzymatic activities of PMTs from all three subfamilies [13,20]. Mutants were grown in the presence of 1 µM of inhibitor on solid growth medium overnight. Growth areas of deletion and DAmP mutants in the presence of inhibitor were compared to untreated controls and expressed in percent. Among almost 6000 mutants, 104 showed a severe decrease in growth (by 75% or more) in the presence of the PMT-inhibitor (Supplementary Table S1) and were further analyzed by GeneCodis [21,22,23]. Functional classification of genes corresponding to the mutants led to the identification of 11 enriched biological processes (Figure 1). Manual verification of the results, using the *Saccharomyces* Genome Database [24], introduced minor changes in the gene assignment to specific gene ontology (GO) terms and the addition of two additional terms: “cell wall integrity pathway” and “calcineurin signaling pathway” (Table 1). Moreover, the GO terms “pexophagy” and “response to acid” were removed. The term “pexophagy” contained exclusively mutants of genes encoding for proteins involved in CWIP. The term “response to acid” enclosed mutants of CWIP components and *RGD1*, encoding for a Ras homologue (Rho) GTPase-activating protein involved in the organization of the actin cytoskeleton [25].

Cellular component GO classification showed that the most affected mutants were enriched in genes encoding for proteins localized to the ER, the plasma membrane and the site of polarized growth. With respect to molecular function, they were enriched in transferase, Rho GTPase and kinase activities [26]. GO terms enriched in the aspect of cellular component and molecular function strongly correlate with subcellular localization and molecular activities of the proteins encoded by the genes enriched among the mutants in the biological process ontology.

Among the enriched biological processes, we found “protein *O*-linked glycosylation”, the process directly targeted by the used drug, confirming the quality of the data. This group includes mutants of individual protein *O*-mannosyltransferases (*PMT1*, *PMT2*, *PMT6* and the putative *O*-mannosyltransferase *PMT7*), of phosphoglucose isomerase (*PGI1*), which is crucial for the biosynthesis of the mannose donor molecules GDP-mannose and Dol-P-Man, and the Golgi GDP-mannose transporter (*VRG4*) affecting the elongation of *N*- and *O*-linked glycans in the Golgi apparatus (Table 1).

The most common GO terms are related to cell wall organization, biogenesis and stress response (Table 1). We found among the mutant hits multiple factors involved in the signal transduction within the CWIP (reviewed in [14]). This pathway involves highly *O*-mannosylated cell surface sensors coupled to the small G-protein Rho1, which activates the protein kinase Pkc1. In turn, Pkc1 triggers the mitogen-activated protein kinase (MAPK) cascade composed of Bck1, redundant Mkk1 and Mkk2 and Slt2/Mpk1. The latter MAPK stimulates transcription factors that activate the expression of the target genes involved in cell wall synthesis and organization. In addition to the sensors (*MID2*, *WSC1*), factors regulating Rho1 (*SAC7*, *BEM2*, *ACK1*), the MAP-kinase relay (*BCK1*, *MKK1*, *SLT2*) and a transcription activator (*RLM1*; 74% growth inhibition, Supplementary Table S1), as well as cell polarity factors, i.e., recruiting the MAP-kinase module to sites of polarized growth (*SPA2*, *BEM1*), are among the major hits of our screening (Table 1). We also identified two members (*SPT3*, *SPT8*) and a partner (*CTI6*) of SAGA (Supplementary Table S1), a multi-subunit complex regulating gene expression in response to CWIP activation by its histone acetylase activity [27,28].

Further, our screen revealed components of the calcineurin signaling pathway (reviewed in [29]) (Table 1). Calcineurin is a protein phosphatase regulated by calcium ions. During external stresses, calcium concentration in the cytoplasm increases and triggers the enzymatic activity of calcineurin. Calcineurin then activates the transcription factor Crz1, which targets the genes promoting the cell survival by different mechanisms including cell wall biosynthesis. We identified among the mutant hits a high-affinity calcium channel (*CCH1*), calcineurin catalytic and regulatory subunits (*CMP2*, *CNB1*), as well as *SKN7*, which was shown to stabilize Crz1 [30]. Upon activation of calcineurin signaling, CWIP (whose hyperactivation is deleterious [31]) is downregulated by the Slt2-inhibiting protein tyrosine phosphatase 2 [32,33] (*PTP2*, Supplementary Table S1), highlighting the synergistic function of these pathways. The importance of the proper regulation of CWIP upon PMT inhibition is further attested by a negative regulator of Bck1 (*NBP2*, Table 1) [34].

In addition to the components of the signaling pathways regulating cell wall integrity, downstream targets of these pathways are specifically enriched in our screening. Factors involved in cell wall remodeling such as the machinery for chitin synthesis (*CHS1*, *CHS3*, *CHS7*, *SKT5*) or *KRE9*, which is crucial for the assembly of β-1,6-glucan, have been identified (GO terms “fungal-type cell wall organization” and “cellular cell wall organization”, Table 1).

Furthermore, glycosylation pathways important for proper folding, ER-quality control, maturation, sorting from the ER to the cell wall and function of cell wall proteins are enriched in the screening. These include *O*-mannosylation (see above), the assembly and protein transfer of *N*-linked glycans in the ER (i.e., *ALG3*, *WBP1*, *OST3*), as well as the biosynthesis and maturation of GPI anchors (i.e., *ERI1*, *GUP1*, *TED1*) (Table 1). Finally, when *O*-mannosylation is decreased, cell growth depends on the unfolded protein response (*HAC1*, *IRE1*; Table 1), which maintains ER functionality during exposure to protein folding, secretion and cell wall stress (reviewed in [35,36]).

Additional GO terms enriched in our analysis are response to osmotic stress and establishment of cell polarity (Table 1). Among hits not assigned to any enriched GO term, we found multiple genes encoding for proteins involved in ubiquitin-mediated protein degradation (*RPT6*, *DOA1*, *RPL40B*, *POC4*, *UBX2*, *RPN10*) and in different protein transport processes (*BST1*, *ERV25*, *SEC72*, *SXM1*, SYT1, *TOM5*, *VID22*) (Supplementary Table S1).

### 2.2. Pmt2 N-Glycans are Ost3 Specific

**Figure 2:**
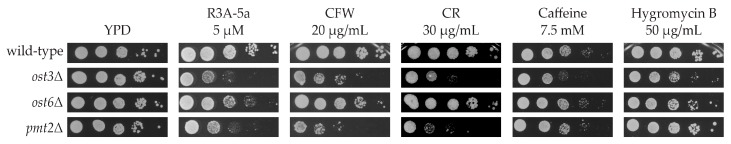
Analysis of cell growth in the presence of different drugs. Three microliters of serial 10× dilutions of cells starting with 6 × 10^5^ cells (left) were spotted on solid media containing no drug (YPD), PMT-inhibitor (R3A-5a), calcofluor white (CFW), Congo red (CR), caffeine or hygromycin B.

Our previous work indicated that *O*-mannosylation and *N*-glycosylation are interconnected at various levels [5,6,7,13]. Here, we identified mutants of two subunits of the OST complex, Wbp1 and Ost3, which displayed a negative growth phenotype when *O*-mannosylation was impaired (Table 1). Ost3 and its homolog Ost6 are alternatively present in the OST complex influencing its substrate specificity [37]. To verify the different sensitivities of *ost3*Δ and *ost6*Δ strains towards the PMT-inhibitor, we performed spotting assays (Figure 2). In the presence of 5 μM R3A-5a on solid media, the deletion of *OST6* resulted only in mild sensitivity to the drug when compared to the wild-type, while the growth of the *ost3*Δ strain was as severely impaired, as the growth of *pmt2*Δ cells. Similar performance was observed in the presence of agents known to decrease the growth of mutants with cell wall defects [38,39,40] (Figure 2). Interestingly, in the case of agents directly targeting cell wall glycans (Congo red and calcofluor white [41]), the growth of the *ost3*Δ mutant was impaired to the same extent as the growth of *pmt2*Δ cells.

**Figure 3:**
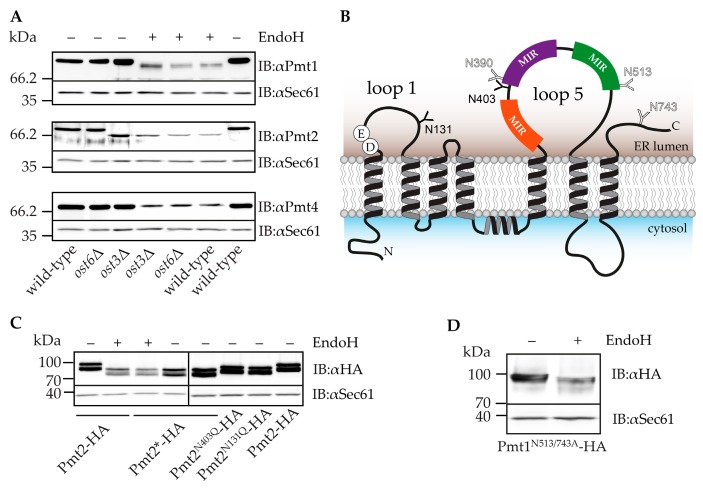
(**A**) Analysis of Pmt1, Pmt2 and Pmt4 *N*-glycosylation site usage in wild-type, *ost3*Δ and *ost6*Δ. Total membranes were prepared from corresponding strains, and 20 µg of proteins were loaded per lane on 8% polyacrylamide gel. Proteins were separated by SDS-PAGE and analyzed by immunoblot. The ER translocation channel Sec61 served as a loading control. *N*-glycans were removed by endoglycosidase H (EndoH) treatment of total membranes prior to loading on the gel. (**B**) Topology model of PMTs with marked *N*-glycosylation sites of Pmt2 (**𝐘**) and Pmt1 (𝕐). The conserved DE motif, crucial for the activity of the complex [10], is indicated in loop 1. Three MIR (mannosyl transferase, inositol triphosphate and ryanodine receptor) domains placed in loop 5, which are essential for enzymatic activity in vitro and in vivo, but that do not affect binding of a peptide substrate [10,42], are colored in orange, purple and green, respectively. (**C**) Analysis of Pmt2 *N*-glycosylation site usage. Samples were prepared from strains EZY50 (Pmt2-HA), EZY51 (Pmt2^N131Q^-HA), EZY52 (Pmt2^N403Q^-HA) and EZY53 (Pmt2*-HA) and analyzed as described for (**A**). The HA-tagged version of Pmt2 appears on the immunoblot as a double band, detectable also with a Pmt2-specific antibody [43]. The presence of two bands might be due to a further processing of the protein, different from *N*-glycosylation; the double band is also present after EndoH treatment. (**D**) Analysis of conserved Pmt1 *N*-glycosylation site (N390) usage in *ost3*Δ. Samples were prepared from the EZY88 (Pmt1^N513/743A^-HA) strain and analyzed as described for (**A**), except 7% polyacrylamide gel was used.

The fact that *ost3*Δ but not *ost6*Δ cells are highly sensitive towards the PMT-inhibitor prompted us to put forward the hypothesis that Ost3 might be modifying one of the PMTs in a way that promotes their function. Therefore, we studied the specificity of Ost3 and Ost6 towards the three major PMTs: Pmt1, Pmt2 and Pmt4. Total membranes isolated from wild-type, *ost3*Δ and *ost6*Δ strains were analyzed by SDS-PAGE combined with immunoblot (Figure 3A). The *N*-glycosylation status of membrane proteins was verified by comparing their apparent molecular weight before and after removal of *N*-glycans by endoglycosidase H (EndoH).

The apparent molecular weight of Pmt4 is comparable in wild-type and *ost*Δ mutant strains and not affected by EndoH treatment, showing that Pmt4 does not carry *N*-glycans (Figure 3A, lower panel). Within the Pmt4 sequence, there is only one *N*-glycosylation sequon [44]. However, since the acceptor asparagine is placed four amino acids from the C-terminus of the protein, its usage is highly unlikely [45]. Pmt1 contains three *N*-glycosylation sites, which were previously proven to be modified [42]. Two of them (N390 and N513) are located in loop 5 and one (N743) in the C-terminal region (Figure 3B). As shown in Figure 3A (upper panel), Pmt1 is *N*-glycosylated to the full extent in the absence of either *OST3* or *OST6*, demonstrating that both OST complexes can act on this membrane protein. In contrast, the apparent molecular weight of Pmt2 is only decreased in *ost3*Δ cells. The molecular mass correlates with the de-glycosylated protein (Figure 3A, middle panel), demonstrating that *N*-glycosylation of Pmt2 is dependent specifically on Ost3. Sequence analysis of Pmt2 revealed four potential *N*-glycosylation sites [44]. Two of the sequons are highly unlikely to be modified since N155 and N641 are placed within the second predicted transmembrane domain and in the loop 6 domain facing the cytosol, respectively. *N*-glycosylation sequons N131 and N403 are located in the ER luminal loop 1 and loop 5 domains, respectively (Figure 3B). To further characterize *N*-glycosylation site usage of Pmt2, we created hemagglutinin-tagged (HA-tagged) versions and mutated *N*-glycosylation sites by changing N131 and N403 to Q simultaneously (Pmt2*-HA) or individually (Pmt2^N131Q^-HA; Pmt2^N403Q^-HA). Mutation of each of these sites resulted in a protein of smaller molecular weight than its wild-type version (Figure 3C), proving that both sites are modified. Further, simultaneous mutation of N131 and N403 resulted in a protein of the same aberrant molecular mass as wild-type de-glycosylated Pmt2, demonstrating that N131 and N403 are the only modified *N*-glycosylation sequons within Pmt2.

The Pmt2 *N*-glycosylation site in loop 5 is conserved between Pmt1 (N390) and Pmt2 (N403). To directly verify the usage of this site in Pmt1 loop 5 by Ost3, we constructed a version of Pmt1 containing only the *N*-glycosylation site N390 (Pmt1^N513/743A^-HA) and expressed it in *ost3*Δ cells. We analyzed the protein before and after EndoH treatment by immunoblot using 7% polyacrylamide gels for better resolution. As shown in Figure 3D, despite the high sequence similarity between Pmt1 and Pmt2, N390 of Pmt1 is still used in the absence of Ost3.

### 2.3. Impact of N-Glycosylation on Pmt1-Pmt2 Activity

To further specify whether the negative synergistic effect of Ost3 deletion and PMT-specific inhibition is at least partially due to the impact of Ost3-specific *N*-glycans on Pmt2 activity, we investigated the role of these glycans for Pmt2 function. First, we analyzed the stability of non-glycosylated HA-tagged Pmt2 (Pmt2*-HA, see above). For this purpose, we expressed Pmt2 wild-type or mutant protein in a *pmt2*Δ strain from a centromeric plasmid and analyzed membrane proteins by immunoblot. Both Pmt2 versions were equally abundant at the steady state level and equally stable in the cell for up to five hours when protein biosynthesis was blocked by cycloheximide [46].

**Figure 4:**
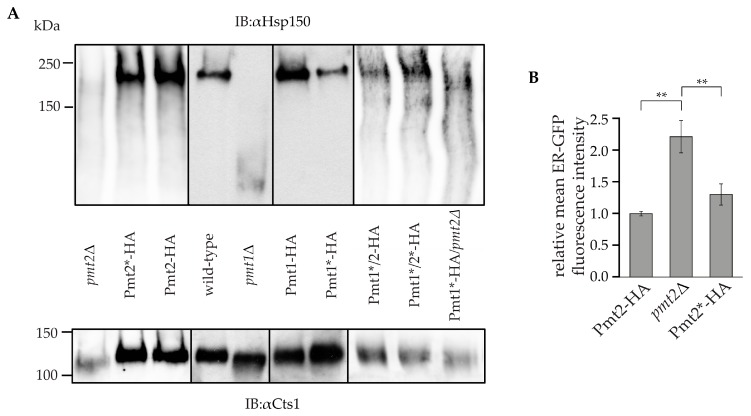
Functional analysis of Pmt2*-HA. (**A**) Analysis of in vivo *O*-mannosylation status of Pmt1-Pmt2 substrates: heat shock protein 150 (Hsp150) and chitinase (Cts1). Samples prepared from strains EZY48 (*pmt2*Δ), EZY50 (Pmt2-HA), EZY53 (Pmt2*-HA), SEY6210 (wild-type), *pmt1*Δ, JHY1 (Pmt1-HA), MLY67 (Pmt1*-HA), EZY66 (Pmt1*-HA/*pmt2*Δ), EZY67 (Pmt1*/2-HA) and EZY68 (Pmt1*/2*-HA) were separated on 6% polyacrylamide gels by SDS-PAGE and analyzed by immunoblot. (**B**) Analysis of ER-GFP fluorescence. Cells from strains EZY54 (*pmt2*Δ), EZY55 (Pmt2-HA) and EZY58 (Pmt2*-HA) were grown to the mid-log phase in liquid medium and analyzed by FACS. The mean value of ER-GFP fluorescence in EZY55 was used as a reference and set to one. The graph shows the fold change in mean values (*n* = 3) ± SD. The results were evaluated by the Tukey HSD test. ** *p*-value < 0.01.

To characterize *O*-mannosyltransferase activity of non-glycosylated Pmt2, we analyzed the in vivo *O*-mannosylation status of two known Pmt1-Pmt2 substrates: heat shock protein 150 (Hsp150) and chitinase (Cts1) [47]. These secreted *O*-mannosylated proteins are not *N*-glycosylated [48,49]. When isolated from *pmt2*Δ cells expressing the wild-type version of Pmt2, Cts1 and Hsp150 show an aberrant molecular mass of 120 and of above 150 kDa, respectively (Figure 4A). Upon perturbation of *O*-mannosylation (Figure 4A, *pmt1*Δ and *pmt2*Δ), both proteins shift to lower molecular masses. However, the absence of *N*-glycans in Pmt2 did not significantly affect the *O*-mannosylation status of these substrate proteins (Figure 4A).

Pmt2 acts in a complex with Pmt1 which is also *N*-glycosylated (Figure 3). To elucidate whether the absence of *N*-glycans of both complex partners has a more pronounced effect on the complex activity, we analyzed the *O*-mannosylation status of Cts1 and Hsp150 from cells expressing non-glycosylated Pmt1 (Pmt1*-HA) in combination with wild-type or non-glycosylated Pmt2. Again, no significant differences of the *O*-mannosylation status of the substrate proteins were observed when compared to wild-type conditions (Figure 4A).

Next, we examined the importance of Pmt2 *N*-glycans for its function in the ER quality control by monitoring the *O*-mannosylation status of the known unfolded protein *O*-mannosylation substrate: ER-GFP. ER-GFP is a version of GFP targeted to the ER via the Kar2 signal peptide and retained there by an HDEL sequence. The *O*-mannosylation of GFP in the ER environment by the Pmt1-Pmt2 complex prevents it from reaching its final conformation and therefore also extinguishes its ability to exhibit fluorescence. When Pmt1 or Pmt2 are deleted, *O*-mannosylation of ER-GFP is decreased leading to better folding and increased fluorescence of ER-GFP, which can be measured by flow cytometry [19]. Upon expression of wild-type or non-glycosylated Pmt2, ER-GFP fluorescence is significantly reduced (Figure 4B), showing that *N*-glycans of Pmt2 are also dispensable for its in vivo activity with respect to unfolded protein *O*-mannosylation.

Manya et al. showed that *N*-glycans of human PMTs are crucial for their activity in vitro [50]. If the effect of Pmt2 *N*-glycans on its function is more subtle, it may be concealed in vivo due to compensatory mechanisms in the cell. Thus, we analyzed in vitro *O*-mannosyltransferase activity of membranes from cells expressing Pmt2 lacking *N*-glycans. We observed that the mean transfer of [^3^H]mannose from Dol-P-[^3^H]Man to the acceptor YATAV peptide by non-glycosylated Pmt2 was reduced in comparison to the wild-type protein (Table 2). Despite the variations between replicates, activities obtained for the Pmt2 *N*-glycan mutant never reached the mean transfer observed for the wild-type enzyme, suggesting a subtle impact of *N*-glycosylation on Pmt2 activity in vitro.

## 3. Discussion

The yeast cell wall is an external envelope defining cell shape and structural integrity. The cell wall is formed by an elastic framework of polysaccharides (β-1,3- and β-1,6-glucans and chitin) to which various glycosylated proteins are linked (reviewed in [51]). Most cell wall proteins are highly *N*-glycosylated, *O*-mannosylated or both, and approximately half of all cell wall annotated proteins are known or predicted GPI-anchored proteins [7]. At the cell surface, these proteins play key roles in cell wall synthesis and structure, morphogenesis and cell adhesion (reviewed in [51]). *N*- and *O*-linked glycans function in the quality control of glycoproteins in the ER and may affect the targeting, transport and functionality of cell wall proteins (reviewed in [52,53,54]). Consequently, defects in *O*- and *N*-glycosylation result in various cell wall-related phenotypes [1,12,55,56]. Similarly, when GPI anchor synthesis is impaired, cell wall proteins may accumulate in the ER or may be released to the medium [57], resulting in cell wall defects. Our screening suggests substantial interconnection between these three types of glycosylation, each one essential for cell viability, which may take place at multiple levels.

GPI anchors are synthesized in the ER via a multistep pathway depending on more than 20 mainly essential gene products (reviewed in [58]). The completed GPI anchor is attached to proteins entering the ER, which contain a C-terminal GPI signal anchor sequence. In yeast, further sequential remodeling of both glycan and lipid moieties of the protein-linked GPI anchor takes place inside the ER. After that, GPI-anchored proteins bind to the receptor p24 complex for vesicular sorting [59]. At the cell surface, the majority of GPI-anchored proteins are further processed and covalently linked to β-1,6-glucan of the cell wall through a remnant of the GPI anchor [51]. The present study demonstrates that mutants affecting the biosynthesis (*MDC4*, *ERI1*, *CWH43*, *GUP1*, *GWT1*) or remodeling (*BST1*, *LAS21*, *TED1*) of GPI anchors, the export of GPI-anchored proteins from the ER (*ERV25*), as well as cell wall β-1,6-glucan assembly (*KRE9*, Table 1; *KRE5* and *KRE6*: 69% and 67% growth inhibition, Supplementary Table S1) are especially sensitive towards the PMT-inhibitor (Table 1 and Supplementary Table S1). The interlocking of these steps may take place on different levels. The recently-established yeast *O*-mannose glycoproteome revealed that 78% of all known and predicted GPI-anchored proteins, as well as most proteins necessary for β-1,6-glucan assembly (including the essential gene product of *KRE9*) are extensively *O*-mannosylated [7]. As demonstrated for individual examples, *O*-mannosylation may impact, e.g., protein stability, localization and function [60,61,62,63]. Based on the physical interaction between Pmt1-Pmt2 complexes and p24 proteins (e.g., Erv25), as well as the observed impact of Pmt1/Pmt2-mediated *O*-mannosylation on the ER export of the GPI-anchored protein Gas1, it was suggested that PMTs help to withhold GPI-anchored proteins in the ER until they are competent for export [17]. Reduced activity of PMTs might lead to accumulation of GPI-anchored proteins, causing severe ER stress. Furthermore, *O*-mannosylation of the GPI anchor remodeling enzyme Ted1 and of the p24 complex compounds (Erv25, Emp24) might significantly affect their functionality [7]. Impairment of *O*-mannosylation coupled with reduced efficiency of GPI-anchored protein maturation may bring the GPI anchor machinery below a viability threshold, which might be the reason for the synthetic effect we see between these two pathways.

Protein *N*-glycosylation starts in the ER with the assembly of the *N*-glycan precursor Glc3Man9GlcNAc2 on dolichol phosphate (reviewed in [64]). This complex process requires nucleotide and dolichol phosphate-activated monosaccharides, as well as several ER-resident glycosyltransferases (ALGs). The core oligosaccharide is transferred en bloc by the OST membrane protein complex to asparagine in the sequon N-X-S/T of proteins entering the ER. In yeast, the OST complex contains nine protein subunits (Ost1 to Ost6, Stt3, Swp1, Wbp1) and interacts with the translocon complex [65,66]. In the ER, protein linked *N*-glycans are trimmed and constitute important players in ER protein quality control (reviewed in [52]). While glycoproteins travel through the Golgi apparatus to their final destinations, *N*-glycans are further modified by different glycosyltransferase complexes [56,67,68]. Several lines of evidence suggest that *N*-glycosylation and *O*-mannosylation are tightly interconnected. Pmt1-Pmt2 complexes physically interact with the Sec61 translocon and the OST complex [5]. At the translocon, PMTs and OST can even compete for glycosylation sites in vitro and in vivo [5,6,7]. Further, transcriptome analysis under blocked *O*-mannosylation even suggested that outer chain structures of *N*-linked and *O*-mannosyl glycans may, at least in part, functionally replace each other [13]. Hence, mutants with defects in the assembly of the lipid-linked precursor glycans (*ALG6*, *ALG7*), in OST (*WBP1*, *OST3*) and Sec61 complexes (*SEC72*, *SEC61*: 74% growth inhibition; Supplementary Table S1), in trimming reactions of *N*-linked glycans in the ER (*CWH43*), as well as the transport of GDP-mannose into the Golgi apparatus (*VRG4*) are especially sensitive towards PMT-inhibition (Table 1). A further level of interconnection between *O*-mannosylation and *N*-glycosylation became obvious due to the finding that PMTs are per se *N*-glycosylated. For human PMTs, the role of *N*-glycans on protein hydrophobicity and consequently on their enzymatic activity in vitro has been demonstrated [50]. Thus, we drew special attention to the different OST complexes (containing either Ost3 or Ost6) and their effect on yeast PMTs. Ost3 and Ost6 represent non-essential, alternative members of the OST complex, which act on different subsets of target sites [37,69]. In contrast to previous data [70], we found that mainly Ost3 is important for cell wall integrity (Figure 2). In agreement with our data, a more recent study revealed that the majority of *N*-glycosylation sites in cell wall proteins are targeted by the Ost3-containing OST complex [71]. Our finding that exclusively the Ost3-containing OST complex is involved in *N*-glycosylation of Pmt2 (Figure 3) suggested that, in addition to a general reduction of *N*-glycans on cell wall proteins, PMT activity might be affected in the *ost3*Δ mutant. However, under the conditions tested, no major impact of *N*-linked glycans on Pmt1-Pmt2 activity was observed in vivo (Figure 4). More subtle effects on Pmt2 as suggested by in vitro analysis (Table 2) may be masked in vivo by the activity of other PMTs. In the presence of PMT-inhibitor, when other PMTs cannot compensate for Pmt2 function, the reduced *N*-glycosylation indeed might alter the functionality of the protein. Although the *N*-glycosylation site between the first and second MIR domain in loop 5 of Pmt1 and Pmt2 is conserved (Figure 3B), only *N*-glycosylation of Pmt2 depends exclusively on Ost3. Thus, PMTs are well-suited model proteins to analyze differences in substrate requirements between Ost3- and Ost6-specific isoforms of the OST complex, which so far are only poorly defined [72,73].

A major cellular response that ensures survival of yeast cells upon decreased PMT activity is the activation of the biosynthesis of cell wall stress chitin (Table 1), which is in agreement with increased level of chitin in the cell wall of *pmt1pmt2*Δ and *pmt2pmt3*Δ mutants [1]. This response is triggered by the signaling pathways CWIP and calcineurin [74,75,76].

**Figure 5:**
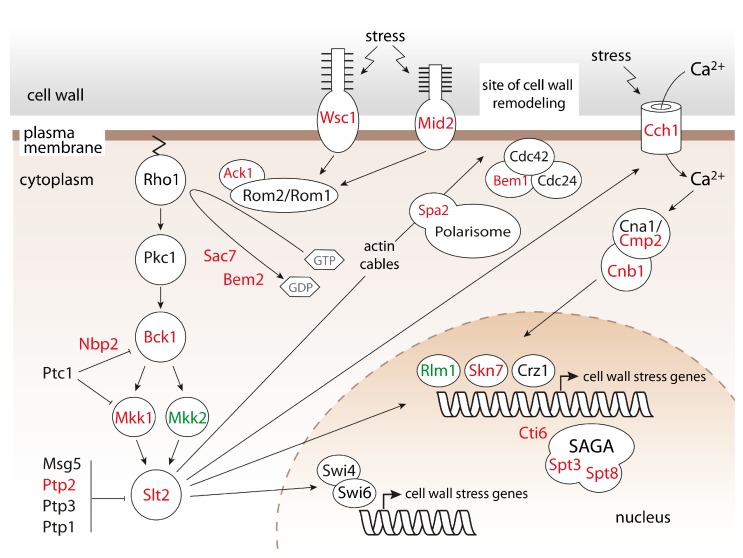
Scheme representing major factors of CWIP and the calcineurin signaling pathway. Impaired *O*-mannosylation results in severe growth defect in the absence of factors depicted in red (growth inhibition of 75% or more) and green (growth inhibition of 74% *mkk2*∆ and 73% *rlm1*∆). Details are described in Section 2.1. Our screening also revealed Ste20 and Tor1, involved in mating/filamentous growth/sterile vegetative growth signaling and in nutrient sensing, respectively (Supplementary Table S1).

In line with our previous findings that *pmt*Δ mutants depend on the activation of CWIP [13,60], the growth of mutants of the major CWIP players and various factors affecting it is impaired upon PMT inhibition (summarized in Figure 5). Our data suggest that both CWIP and the calcineurin pathway are necessary to ensure cell grow when *O*-mannosylation is diminished (Table 1).

During the response to impaired *O*-mannosylation, the unfolded protein response, which is defined by its activators Ire1 and Hac1 (Table 1), plays a prominent role [13]. In yeast, unbalanced ER protein homeostasis is monitored by the transmembrane sensor kinase/endoribonuclease Ire1 (reviewed in [77]). When activated, the cytosolic endoribonuclease domain cleaves the intron of pre-mRNA *HAC1*, thereby enabling translation of this transcription factor and, consequently, upregulation of numerous target genes including ER chaperones [16]. CWIP triggers the unfolded protein response and vice versa, in order to protect yeast cells against the related stress conditions: cell wall and ER stress [36,78,79]. Our previous findings showed that unfolded protein response induction is not defined by just CWIP when *O*-mannosylation is impaired. Unfolded protein response-linked transcriptional response induced by inhibition of PMTs differs from those triggered by constitutive active CWIP or other cell wall mutants. In addition, deletion of *HAC1* in viable *pmt*Δ mutants causes cell death even when cells are osmotically stabilized [13]. In recent years, the multi-faceted impact of *O*-mannosylation on ER homeostasis became obvious. In addition to the effects of PMTs on the synthesis and transport of cell surface proteins, especially GPI-anchored proteins (see above), Pmt1-Pmt2 turned out to be the major *O*-mannosyltransferase modifying unfolded proteins [19]. Furthermore, glycoproteomics revealed *O*-mannosylation of 22% of all proteins curated for ER localization, including chaperones, folding helpers, as well as the unfolded protein response receptor Ire1 [7]. Whether and how these glycans affect the functionality of their carrier proteins and consequently impact on ER homeostasis is still an open question.

Today, the importance of specific types of protein-linked glycans for numerous cellular processes is very well documented in fungi, metazoans and humans. However, in most of the cases, glycoproteins bear more than one type of glycans, and the different glycosylation machineries themselves are glycosylated. Our study revealed that in yeast *O*-mannosylation, *N*-glycosylation and GPI anchor synthesis are interconnected processes that ensure the proper function of the ER and the cell wall. Further unravelling the different levels of this interconnection and the coordinated regulation of the players involved will be a major challenge in the future.

## 4. Materials and Methods

### 4.1. Yeast Strains and Culture Conditions

*Saccharomyces cerevisiae* strains used in this study (apart from the R3A-5a screen, Section 4.2.) are listed in Table 3. Yeast strains were grown in yeast extract-peptone-dextrose (YPD) or synthetic defined medium at 30 °C. Transformations with plasmids pRS415, pRS416, pWX206 [19] and the plasmids described below were performed using the method of Gietz et al. [80]. For genomic integrations, yeast strains were transformed with PCR product following the protocol of Hill et al. [81]. All deletions and genomic integrations were verified by PCR on genomic DNA, prepared according to Looke et al. [82]. Sequences of oligonucleotides are available upon request. All plasmids were checked by DNA sequencing.

To generate *pmt2*Δ strain, *PMT2* gene was knocked out in the BY4741 strain by transformation and homologous recombination with a *loxP-kanMX4-loxP* integration cassette amplified by PCR on pUG6 with oligos 1963 and 1964. Next, the *kanMX4* marker was removed using the Cre-*lox P* recombination system as described in Guldener et al. [86].

To obtain a strain expressing Pmt1 lacking *N*-glycosylation sites in the *pmt2*Δ background, the *pmt1*Δ*pmt2*Δ strain [85] was transformed with a *pmt1-N390A/513/743A^3xHA^::kanMX6* integration cassette amplified by PCR on MLY67 genomic DNA with oligos 1513 and 1516. The resulting strain was then transformed with plasmid pRS416, pEZ78 or pEZ79 to create strains EZY66, EZY67 and EZY68, respectively.

To create plasmid pEZ43 (PMT2^6xHA^), the *PMT2* sequence was subcloned from pVG80 [9] into pRS415 via PstI and SalI restriction sites. *PMT2* point mutations were introduced into pEZ43 via site-directed mutagenesis using recombinant PCR [87]. Plasmids pEZ56 (*pmt2-N131Q^6xHA^*) and pEZ57 (*pmt2-N403Q^6xHA^*) were assembled by generating a PCR fragment with the mutagenic primer pair 2589/2590 or 2594/2593, respectively, in combination with the outer primers 2588 and 2595 and subcloning the resulting fragment into pEZ43 linearized with AfeI and BglII via homologous recombination in yeast. To create plasmid pEZ58 (*pmt2-N131/403Q^6xHA^*), a similar approach was used as for the construction of pEZ56 and pEZ57, with the difference that the PCR fragment was assembled by recombinant PCR with outer primers 2588 and 2595 from two PCR pre-fragments: (1) obtained with the mutagenic primer pair 2589/2590 in combination with primers 2588 and 2592; (2) obtained with the mutagenic primer pair 2594/2593 in combination with primers 2591 and 2595.

To create plasmids pEZ78 and pEZ79, the *PMT2* sequence was subcloned via SalI and SmaI restriction sites into pRS416 from pEZ58 and pEZ43, respectively.

To create plasmid pEZ82 (*pmt1-N513/743A^3xHA^*), we performed ligation of 3 fragments: (1) backbone of pWX206 obtained by restriction digest with BamHI and XbaI; (2) PCR fragment generated with primer pair 2833/2834 on SEY6210 genomic DNA and subsequently digested with BamHI and EcoRI; (3) PCR fragment generated with primer pair 2835/2836 on MLY67 genomic DNA and subsequently digested with EcoRI and XbaI.

### 4.2. R3A-5a Screen

Deletion and DamP mutant collections of *S. cerevisiae* [88] were grown overnight at 30 °C on solid YPD (using 1536-well microplates) in the absence and presence of 1 µM R3A-5a compound. Colony areas were measured from digital images of the plates using Balony software (Barry Young; University of British Columbia, Vancouver, BC, Canada) [89] and compared using Excel (Microsoft; Munich, Germany).

### 4.3. Spotting Assay

Yeast cells were grown to the mid-log phase, harvested and resuspended to a concentration of 6 × 10^5^ cells/mL. Three microliters of initial concentration and 5 serial 10× dilutions were spotted on solid media and incubated at 30 °C for 48 h.

### 4.4. Preparation of Total Membranes and Endoglycosidase H Treatment

Yeast total membranes were prepared from mid-log phase cultures as described previously [11]. Removal of *N*-glycans was performed using EndoH (#P0702; New England Biolabs; Frankfurt/Main, Germany) and supplied buffers according to the protocol provided by the manufacturer with the following modifications. Samples (30 µg of membrane proteins) were denatured at 50 °C and incubated with 250 U of enzyme in the presence of 5 mM phenylmethylsulfonyl fluoride. The reaction was stopped by adding 5 µL of 5× SDS-sample buffer.

### 4.5. Isolation of Heat Shock Protein 150 and Chitinase

Hsp150 was isolated from the culture medium (equivalent to 25 OD of cells) as described previously [48]. Cts1 was precipitated from the medium of stationary phase cultures (equivalent to 5 OD of cells) by adding 100% TCA in acetone (weight/volume) to a final concentration of 15% and incubating on ice for 1 h. Next, the pellets were collected by centrifugation (20,000 rcf 10 min), washed thrice with ice-cold acetone, air-dried for 1 min at room temperature and resuspended in 1× SDS-sample buffer.

### 4.6. SDS-PAGE and Immunoblot

Protein samples were denatured in 1× SDS-sample buffer for 10 min at 50 °C (for detection of PMTs) or 95 °C (for detection of Hsp150 and Cts1), resolved on glycine polyacrylamide gels and transferred to nitrocellulose. Blots were incubated with primary antibodies, listed in Table 4, and horseradish peroxidase-conjugated anti-mouse (dilution 1:10,000; #A9044; Sigma-Aldrich; Steinheim, Germany) or anti-rabbit (dilution 1:5,000; #A6154; Sigma-Aldrich) secondary antibodies. For visualization of protein-antibody complexes we used enhanced chemiluminescence (Amersham ECL Detection Reagents; GE Healthcare; Munich, Germany). 

### 4.7. Flow Cytometry

Cells expressing ER-GFP were grown to the mid-log phase and metabolically inhibited by addition of sodium azide to a final concentration of 100 mM. Cells were gently sonicated, and the GFP fluorescence intensity of 20,000 cells was quantified by flow cytometry using BD FACSCanto^TM^ II (BD Biosciences; Heidelberg, Germany).

### 4.8. In Vitro Mannosyltransferase Activity Assay

In vitro mannosyltransferase activity assay was performed as described previously [11] in the presence of 0.12% Triton X-100 as a detergent and using biotinylated YATAV peptide (Biopolymers Thermo Scientific; Bonn, Germany) as the acceptor substrate.

## Figures and Tables

**Table 1:** List of genes assigned to a particular biological process after manual verification using the *Saccharomyces* Genome Database.

GO Term Biological Process	Gene Names
GPI anchor biosynthetic process	*BST1*, *CWH43*, *ERI1*, *GPI11*, *GUP1*, *GWT1*, *LAS21*, *MCD4*, *TED1*
Response to osmotic stress	*MID2*, *NBP2*, *NHA1*, *RGD1*, *RVS161*, *SKN7*, *STE20*, *WSC1*
ER unfolded protein response	*BCK1*, *HAC1*, *IRE1*, *SLT2*, *VPS74*
Fungal-type cell wall organization	*ACK1*, *CHS1*, *CNB1*, *CWH43*, *IRE1*, *KRE9*, *MID2*, *PMT1*, *PMT2*, *PMT6*, *PMT7*, *SIM1*, *WSC1*
Protein *O*-linked glycosylation	*PGI1*, *PMT1*, *PMT2*, *PMT6*, *PMT7*, *VRG4*
Protein *N*-linked glycosylation	*ALG6*, *ALG7*, *CWH41*, *OST3*, *PGI1*, *ROT2*, *VRG4*, *WBP1*
Establishment of cell polarity	*BCK1*, *BEM1*, *BEM2*, *RGD1*, *SAC7*, *SPA2*, *WSC1*
ER-associated misfolded protein catabolic process	*PMT1*, *PMT2*, *PMT6*, *PMT7*
Cellular cell wall organization	*CHS1*, *CHS3*, *CHS7*, *KRE9*, *MCD4*, *SIM1*, *SKT5*, *TRS65*, *WSC1*
Cell wall integrity pathway	*ACK1*, *BCK1*, *BEM2*, *MID2*, *MKK1*, *SAC7*, *SLT2*, *SPA2*, *WSC1*
Calcineurin signaling pathway	*CCH1*, *CMP2*, *CNB1*

**Table 2:** Mannosyltransferase activity in vitro.

Strain	[^3^H]Man Incorporation (×10^3^ dpm) ± SD	% ± SD
Pmt2-HA	3.909 ± 1.348	100 ± 35
*pmt2*Δ	0.165 ± 0.05	4 ± 1
Pmt2*-HA	2.328 ± 0.625	60 ± 16

Total membrane fractions from strains EZY48 (*pmt2*Δ), EZY50 (Pmt2-HA) and EZY53 (Pmt2*-HA) were tested for Pmt1-Pmt2 in vitro transferase activity with the standard assay containing 31,728 disintegrations per minute (dpm) of Dol-P-[^3^H]Man per reaction. Mean ± SD values of three replicates are shown. *P*-values, calculated using the Tukey HSD test, are: 0.004 for Pmt2-HA vs. *pmt2*Δ; 0.139 for Pmt2-HA vs. Pmt2*-HA; 0.049 for Pmt2*-HA vs. *pmt2*Δ.

**Table 3:** *Saccharomyces cerevisiae* strains.

Strain	Genotype	Reference/Source
BY4741 (wild-type)	*MATa met15-*Δ*0 his3-*Δ*1 leu2-*Δ*0 ura3-*Δ*0*	[83]
*ost3*Δ	BY4741 except *ost3*Δ*::kanMX4*	Euroscarf
*ost6*Δ	BY4741 except *ost6*Δ*::kanMX4*	Euroscarf
*pmt2*Δ	BY4741 except *pmt2*Δ	This study
EZY48	*pmt2*Δ with pRS415	This study
EZY50	*pmt2*Δ with pEZ43	This study
EZY51	*pmt2*Δ with pEZ56	This study
EZY52	*pmt2*Δ with pEZ57	This study
EZY53	*pmt2*Δ with pEZ58	This study
EZY54	EZY48 with pWX206	This study
EZY55	EZY50 with pWX206	This study
EZY58	EZY53 with pWX206	This study
SEY6210	*MATα lys2-801 his3-*Δ*200 leu2-3,112 trp1-*Δ*901 ura3-52 suc2-*Δ*9*	[84]
*pmt1*Δ	SEY6210 except *pmt1::HIS3*	[85]
EZY66	SEY6210 except *pmt2::LEU2 pmt1-N390A/N513A/N743A-3xHA::kanMX6* and with pRS416	This study
EZY67	SEY6210 except *pmt2::LEU2 pmt1-N390A/N513A/N743A-3xHA::kanMX6* and with pEZ79	This study
EZY68	SEY6210 except *pmt2::LEU2 pmt1-N390A/N513A/N743A-3xHA::kanMX6* and with pEZ78	This study
EZY88	*ost3*Δ with pEZ82	This study
JHY1	SEY6210 except *PMT1-3xHA::kanMX6*	[5]
MLY67	SEY6210 except *pmt1-N390A/N513A/N743A-3xHA::kanMX6*	[5]

**Table 4:** Primary antibodies used in this study.

Name	Description	Reference
αPmt1	Rabbit; 1:2500	[90]
αPmt2	Rabbit; 1:2500	[91]
αPmt4	Rabbit; 1:100	[9]
αHA	Mouse; 1:10,000; 16B12	#MMS-101R; Covance
αSec61	Rabbit; 1:2500	Gift from Karin Römisch
αHsp150	Rabbit; 1:2500	[48]
αCts1	Rabbit; 1:1000	[92]

## Supplementary Materials

Supplementary materials can be found at www.mdpi.com/1422-0067/18/6/1226/s1.

## Abbreviations

ER	endoplasmic reticulum
Dol-P-Man	dolichol phosphate-activated mannose
PMT	protein *O*-mannosyltransferase
OST	oligosaccharyltransferase
GDP-mannose	guanosine diphosphate-activated mannose
CWIP	cell wall integrity pathway
GPI	glycophosphatidylinositol
GFP	green fluorescent protein
GO	gene ontology
EndoH	endoglycosidase H
HA	hemagglutinin
